# SARS‐CoV‐2 environmental contamination associated with persistently infected COVID‐19 patients

**DOI:** 10.1111/irv.12783

**Published:** 2020-07-12

**Authors:** Hui Lei, Feng Ye, Xiaoqing Liu, Zhenting Huang, Shiman Ling, Zhanpeng Jiang, Jing Cheng, Xiaoqun Huang, Qiubao Wu, Shiguan Wu, Yanmin Xie, Cheng Xiao, Dan Ye, Zifeng Yang, Yimin Li, Nancy H. L. Leung, Benjamin J. Cowling, Jianxing He, Sook‐San Wong, Mark Zanin

**Affiliations:** ^1^ Guangzhou Medical University Guangzhou China; ^2^ State Key Laboratory of Respiratory Diseases National Clinical Research Center for Respiratory Disease Guangzhou China; ^3^ Department of Pulmonary and Critical Care Medicine The First Affiliated Hospital of Guangzhou Medical University Guangzhou China; ^4^ Department of Intensive Care The First Affiliated Hospital of Guangzhou Medical University Guangzhou China; ^5^ WHO Collaborating Centre for Infectious Disease Epidemiology and Control School of Public Health The University of Hong Kong Hong Kong SAR China; ^6^ Infection Control The First Affiliated Hospital of Guangzhou Medical University Guangzhou China; ^7^ Guangzhou Institute of Respiratory Health Guangzhou China; ^8^ Macau University of Science and Technology Macau SAR China; ^9^ Department of Thoracic Oncology and Surgery The First Affiliated Hospital of Guangzhou Medical University Guangzhou China

**Keywords:** coronavirus, COVID‐19, intensive care unit, SARS‐CoV‐2, transmission

## Abstract

**Background:**

Severe COVID‐19 patients typically test positive for SARS‐CoV‐2 RNA for extended periods of time, even after recovery from severe disease. Due to the timeframe involved, these patients may have developed humoral immunity to SARS‐CoV‐2 while still testing positive for viral RNA in swabs. Data are lacking on exposure risks in these situations. Here, we studied SARS‐CoV‐2 environmental contamination in an ICU and an isolation ward caring for such COVID‐19 patients.

**Methods:**

We collected air and surface samples in a hospital caring for critical and severe COVID‐19 cases from common areas and areas proximal to patients.

**Results:**

Of the 218 ICU samples, an air sample contained SARS‐CoV‐2 RNA. Of the 182 isolation ward samples, nine contained SARS‐CoV‐2 RNA. These were collected from a facemask, the floor, mobile phones, and the air in the patient room and bathroom. Serum antibodies against SARS‐CoV‐2 were detected in these patients at the beginning of the study.

**Conclusions:**

While there is a perception of increased risk in the ICU, our study demonstrates that isolation wards may pose greater risks to healthcare workers and exposure risks remain with clinically improved patients, weeks after their initial diagnoses. As these patients had serum antibodies, further studies may be warranted to study the utility of serum antibodies as a surrogate of viral clearance in allowing people to return to work. We recommend continued vigilance even with patients who appear to have recovered from COVID‐19.

## 1 BACKGROUND

The outbreak of coronavirus disease 2019 (COVID‐19) has strained the capacity of hospitals worldwide, placing healthcare workers at significant risk of exposure. Air and surface contamination with SARS‐CoV‐2 has been detected in hospital settings where newly diagnosed COVID‐19 patients are cared for.^1^, ^2^, ^3^ SARS‐CoV‐2 has also been shown to have a prolonged presence in saliva and stool samples and an environmental stability greater than SARS‐CoV‐2 on surfaces.^4^, ^5^, ^6^, ^7^ Therefore, the risks of nosocomial infections are likely significant.

COVID‐19 patients typically test positive for SARS‐CoV‐2 RNA for extended periods of time, weeks in some cases, necessitating prolonged hospitalization or isolation.^8^, ^9^ Patients who have recovered from severe COVID‐19 can also continue to test positive. Since these patients have been hospitalized for extended periods, it is possible that they have developed humoral immunity to SARS‐CoV‐2 while still testing positive for viral RNA in swabs. The extent of environmental contamination by these patients in healthcare settings is unknown but these data are particularly relevant to inform measures to prevent exposure of healthcare workers. They are also relevant due to the considerations of using the presence of serum antibodies as a surrogate marker of viral clearance in allowing people to return to work. Therefore, it is important to determine whether environmental contamination with SARS‐CoV‐2 can still be associated with patients with serum antibodies.

To address these concerns, we collected air and surface samples from the intensive care unit (ICU) and an isolation ward of The First Affiliated Hospital of Guangzhou Medical University (FAHGMU), which is a designated hospital for the treatment of critical and severe COVID‐19 pneumonia cases in Guangdong Province, a large province in southern China. Two air samplers were used: a sampler developed by the US National Institute of Occupational Safety and Health (NIOSH) that fractionates airborne particles into three size fractions and a cyclonic aerosol particle liquid concentrator. Overall, environmental contamination in the ICU was minimal. Environmental contamination was greater in the isolation ward, in which SARS‐CoV‐2 RNA was detected in multiple samples, including air samples taken in the patient room and bathroom. All patients in this study have serum IgG titers against SARS‐CoV‐2. Therefore, COVID‐19 patients and individuals that have recovered from severe COVID‐19 could still be shedding virus into the air and environment weeks after illness onset.

## 2 METHODS

### 2.1 Collection of surface samples

Surface samples were collected according to the “World Health Organization Surface sampling of MERS‐CoV in health care settings, June 2019”.^10^ Samples were collected using 15‐cm sterile flocked plastic swabs (Shenzhen Mairuikelin Company). Swabs were wetted with viral transport medium (VTM) prior to sample collection and then placed in 15‐mL tubes containing 3 mL VTM.^11^ Samples were collected between 8 am and 11 am.

In the ICU, swabs were taken from areas proximal to four patients showing the highest viral loads by quantitative RT‐PCR prior to sampling and in areas used by healthcare workers. The locations of swabs taken from patient‐specific areas were the floor less than one meter away from patient head, the bed rail, the patient's clothing, the bedsheet, the control panel of the ventilator, and the ventilator outlet valve (samples E01 to E06, respectively). The locations of swabs taken from areas not associated with individual patients were the changing room door handle, the floor of changing room, the faucets at the handwashing station, and the keyboard of shared computer (samples E07 to E10, respectively) (Table 1).

**Table 1:** Environmental and air samples taken from the intensive care unit and isolation ward of The First Affiliated Hospital of Guangzhou Medical University

Intensive care unit		Isolation ward	
Sample ID	Location	Sample ID	Location
**ENVIRONMENTAL SAMPLES**			
Area near the patient		Patient room	
E01	Floor < 1 m from patient head	E01	Floor < 1 m from patient head
E02	Bed rail	E02	Floor > 1 m from patient head
E03	Patient clothing	E03	Bed rail
E04	Bedsheet	E04	Bedside table
E05	Control panel of ventilator	E05	Mobile phone
E06	Ventilator outlet valve	E06	Bedsheet
		E07	Patient mask
		E08	Television remote control
		Patient bathroom	
		E09	Toilet
		E10	Bathroom door handle
		E11	Sink faucet handles
Areas used by healthcare workers			
E07	Changing room door handle	E12	Door handle
E08	Floor of changing room	E13	Floor
E09	Faucets at handwashing station	E14	Handle of mop used by the cleaning staff after cleaning
E10	Keyboard of shared mobile computer (on cart)		
**AIR SAMPLES**			
Area near the patient		Area near the patient	
N01	NIOSH air sampler	N01^a^	NIOSH air sampler
D01	DingBlue air sampler #1	D01^a^	DingBlue air sampler #1
		Patient bathroom	
		N02^a^	NIOSH air sampler
		D02^b^	DingBlue air sampler #1
		D03^c^	DingBlue air sampler #2

^a^ D01 and N01 were collected in the patient room on two consecutive days; then, the samplers were moved to the bathroom.

^b^ D02 was collected when the patient used the toilet.

^c^ D03 was collected one hour after the patient used the toilet.

In the isolation ward, patients were placed in separate rooms with their own bathrooms. Swabs were taken from the rooms of five patients and the bathrooms of two patients. Patients with the highest viral loads in respiratory or stool samples prior to sampling were selected. The locations of swabs taken from patient rooms were the floor less than one meter away from patient head, the floor greater than one meter away from patient head, the bed rail, the bedside table, the patient's mobile phone, the bedsheet, the patient's facemask, and the television remote control (samples E01 to E08, respectively). The locations of swabs taken from patient bathrooms were the toilet, the bathroom door handle, and the faucet handles on the sink (samples E09 to E11, respectively). It should be noted that squat latrines were in the isolation ward bathrooms (Table 1). The locations of swabs taken from areas not associated with individual patients were the changing room door handle, the floor of changing room, and the cleaner's mop handle (samples E12 to E14, respectively) (Table 1).

### 2.2 Collection of air samples

We collected air samples using two cyclonic sampling devices: a two‐stage cyclonic bioaerosol sampler developed by the NIOSH ^10^, ^11^ (NIOSH, Centers for Disease Control and Prevention) and an aerosol particle liquid concentrator (model W‐15, Beijing DingBlue Technology Co, Ltd). Samples were collected between 8 am and 12 pm.

**Figure 1:**
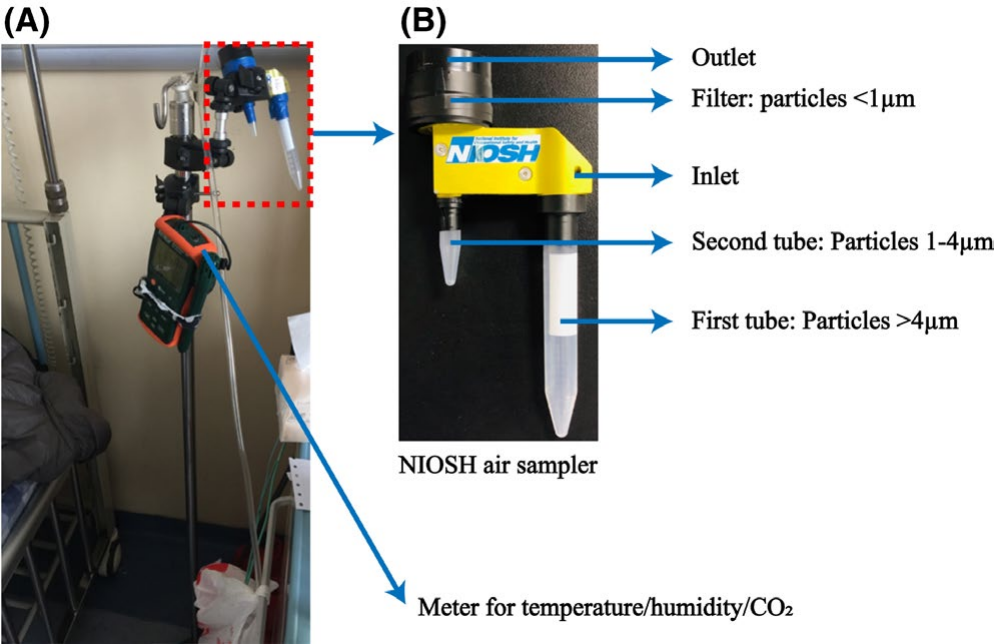
Arrangement of the NIOSH cyclonic bioaerosol sampler. A, Arrangement of the NIOSH sampler on a tripod next to the head of the patient's bed with the temperature, humidity, and carbon dioxide monitor used in this study. B, NIOSH sampler showing collection tubes and collection filter and the particle sizes collected in each

In both the ICU and isolation ward, the NIOSH sampler was placed on a tripod at the head of the bed within one meter of the patient's head at a height of 1.3 m (sample N01, Figure 1 and Table 1). In the isolation ward, the NIOSH sampler was also used in the bathroom by mounting it on an infusion support near the sink, less than one meter from the toilet (sample N02, Table 1). A portable analyzer that recorded temperature and humidity was also mounted on the tripod. Air was collected for four hours continuously at a flow rate of 3.5 L/min into three size fractions: >4 μm (collected in a 15‐mL tube), 1‐4 μm (collected in a 1.5‐mL tube), and <1 μm (collected in a polytetrafluoroethylene (PTFE) membrane filter with 3.0 μm pore size). After each collection, the 15‐mL and 1.5‐mL tubes were detached and 1 mL of VTM was added. The filter was removed and immersed in 1 mL VTM.

The DingBlue sampler was placed at the head of the bed within one meter of the patient's head on the opposite side of the bed to the NIOSH sampler (sample D01). In the isolation ward, the DingBlue samplers were also used to collect air samples from the patient bathroom (samples D02 and D03, Table 1). The sampler was placed on the bathroom sink at a distance of less than one meter from the toilet. Air samples were collected at a flow rate of 14 L/min for 30 minutes into a 5‐mL tube containing 3 mL VTM. Air samples were collected in the bathroom by instructing the patient to turn on the DingBlue air sampler before using the toilet. Medical staff would then collect the sample from the machine after sampling. Other air samples were collected by medical staff.

### 2.3 RNA extraction and quantitative RT‐PCR

All liquid samples were subjected to 30‐min heat inactivation at 56°C prior to RNA extraction as part of national biosafety requirement. RNA was extracted from 0.28 mL of the VTM containing the air and surface samples using the QIAGEN vRNA mini kit (QIAGEN) according to the manufacturer's instructions. RNA samples were screened for the presence of SARS‐CoV‐2 RNA encoding the ORF‐1 or N genes using the “New Coronavirus 2019‐nCoV nucleic acid detection kit (Fluorescence PCR method)” (Sansure Biotech Inc) and an ABI 7500 real‐time PCR machine (Thermo Scientific). The viral copy numbers in patient specimens were calculated using a standard curve established by the diagnostic laboratory of The First Affiliated Hospital of Guangzhou Medical University.

### 2.4 SARS‐CoV‐2 Spike and Nucleocapsid IgG ELISA

Recombinant SARS‐CoV‐2 spike (S) (encompassing the extracellular domain, S1 and S2 subunits) and nucleocapsid (N) proteins (Sino Biological) were used to coat 96‐well plates at 0.5 μg/mL overnight at 4°C. After washing and blocking, serially diluted sera (at a starting dilution of 1:100) were added to the plate and incubated for two hours at 37°C. Plates were washed, and specific antibodies were detected using an anti‐human IgG horseradish peroxidase‐conjugated secondary antibody (Sigma). Colorimetric reaction was developed using 3,3′,5,5′‐tetramethylbenzidine (TMB) substrate (Gibco Technologies). Reactions were stopped using 0.5 mol/L sulfuric acid and absorbance read at 450 nm. End‐point titers were determined to be the last reciprocal dilution with a positive/negative optical density (OD) ratio ≥ 2. Assay specificity for S and N proteins, tested using non‐COVID‐19 sera (n = 203), was comparable at 97.5% and 97.0%, respectively (data not shown).

### 2.5 Pseudovirus antibody neutralization assay

Neutralizing antibody titers against SARS‐CoV‐2 in patient serum samples were determined using a pseudovirus assay as previously described.^3^ Briefly, SARS‐CoV‐2 pseudoviruses were generated by co‐transfection of a lentiviral packaging plasmid, lentiviral reporter plasmid expressing green fluorescent protein and luciferase, and pcDNA3.1 expression vectors encoding the S proteins of SARS‐CoV‐2. Viral supernatants were collected 48 hours post‐transfection. Neutralization assays were performed by combining pseudoviruses and serum (at a starting dilution of 1:40) and incubation at 37°C for one hour. The serum/virus mixture was then added to 293/hACE2 cells in triplicate wells followed by centrifuging the cells at room temperature at 800 rpm for one hour and then incubation for 48 hours at 37°C (5% CO_2_). Luminescence was measured using Steady‐Glo Luciferase Assay System (Promega) and a BioTek Cytation 5 imaging reader (BioTek).

## 3 RESULTS

### 3.1 Minimal environmental contamination with SARS‐CoV‐2 RNA was detected in the ICU

**Figure 2:**
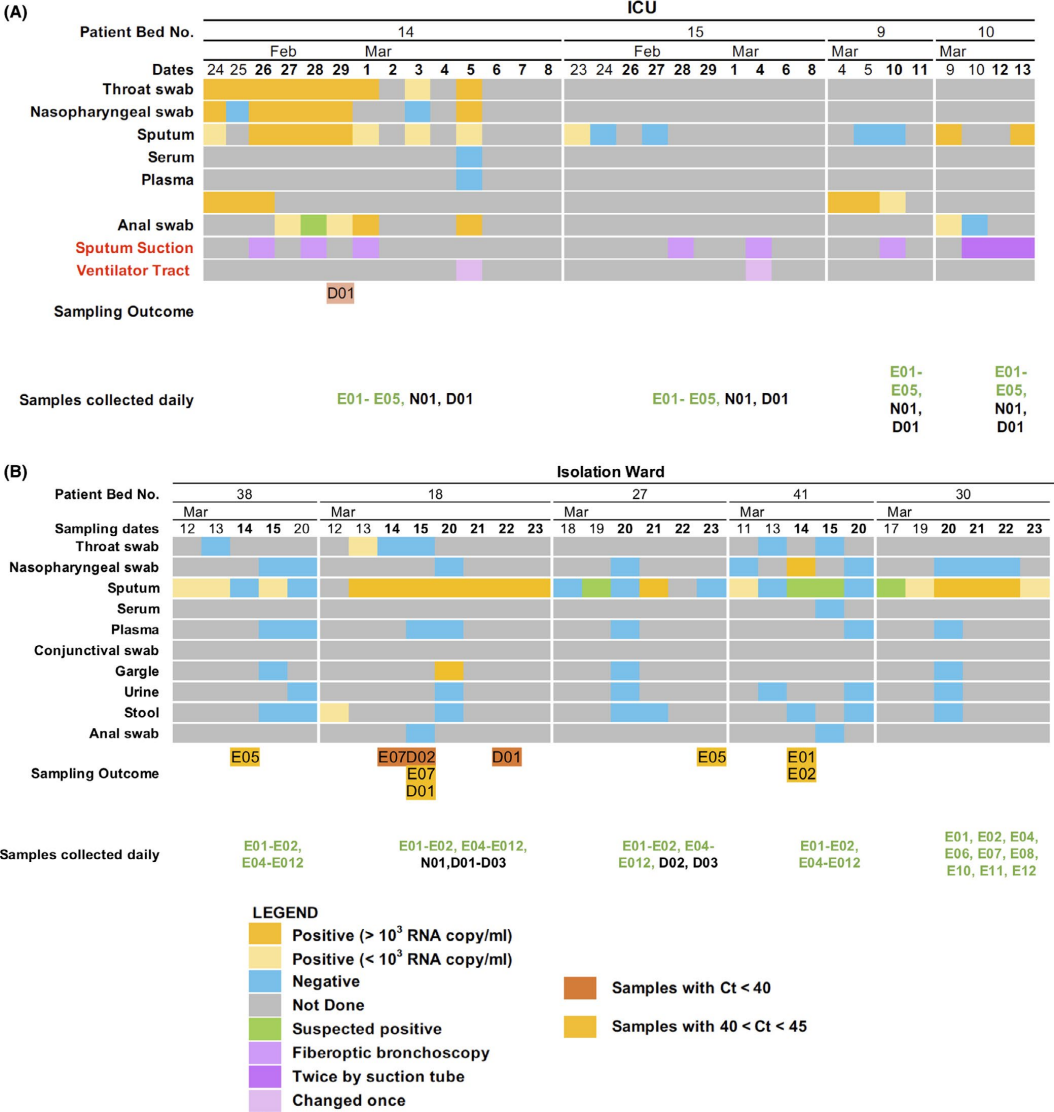
Summary of viral loads in patient swabs, clinical care procedures performed during sampling, and outcomes of environmental sampling. Timeline shows samples collected from patients and testing results for SARS‐CoV‐2 RNA by quantitative real‐time PCR in the (A) intensive care unit and (B) in the isolation ward. Environmental samples that tested positive for SARS‐CoV‐2 RNA by quantitative real‐time PCR are also shown

We collected 218 air and surface samples from the ICU of The FAHGMU over a period of 16 days to determine whether SARS‐CoV‐2 was present in the environment in areas proximal to severely or critically ill COVID‐19 patients and in common areas used by the ICU staff. The airflow in the ICU rooms in which we collected samples was a class 100 000 clean room with laminar flow originating in the ceiling and extracted through wall vents at bed level. Average air changes per hour were 240‐360. The temperature, relative humidity, and concentration of CO_2_ in the ward over the sampling days were relatively constant, at 24.51 ± 0.34°C, 53.28 ± 0.76%, and 813.25 ± 15.96 ppm, respectively. The floor of the ICU is cleaned using chlorine‐containing disinfectant twice a day, at 11 am and 3 pm The furniture and equipment in the ward are also cleaned by wiping with chlorine‐containing disinfectant once a day at 11 am The COVID‐19 patients in the ICU rooms from which we collected samples had been hospitalized for 26, 33, 55, and 47 days when we commenced sampling. The filters used in the respiratory systems were model 1 790 000 Air‐Guard breathing filters (Intersurgical). Three of the four patients were receiving mechanical ventilation and were subjected to procedures previously associated with the generation of aerosols, such as bronchoscopy and intubation, during our sampling period^12^ (Table 2; Figure 2).

**Table 2:** Clinical, hospitalization history, and sampling duration for patients in the intensive care unit of The First Affiliated Hospital of Guangzhou Medical University

Patient Bed No, (age, gender)	Date of symptom onset	Hospitalization history	Clinical presentation upon admission to the intensive care unit (ICU) of The First Affiliated Hospital of Guangzhou Medical University (FAHGMU).	Date of Sampling (duration)	Duration of illness by time of sampling (d)	Ventilation method and clinical care procedures^a^	Samples collected^b^
14, (79, Male)	January 30	First admitted to The Eighth People's Hospital of Guangzhou on January 31. Transferred to the ICU of The FAHGMU on February 6.	COVID‐19 (critical), acute respiratory distress syndrome (severe), septic shock, acute kidney injury, coagulation disorder, liver insufficiency, myocardial damage, multiple organ dysfunction syndrome, diabetes, hypertension, hyperuricemia	February 26 to March 8 (12 d)	27	Tracheotomy ventilation, fiber optic bronchoscopy to clear sputum	E01, E02, E03, E04, E05, N01, D01
15 (55, Female)	January 24	First admitted to The Third Affiliated Hospital of Guangzhou Medical University on January 24. Transferred to The Eighth People's Hospital of Guangzhou on January 26. Transferred to the ICU of The Eighth's People's Hospital of Guangzhou on February 9. Admitted to the ICU of The FAHGMU on February 16.	COVID‐19 (critical), acute respiratory distress syndrome (severe), respiratory failure, sepsis	February 26 to March 1 (5 d) and March 4, 6, and 8 (3 d)	33	Intubated ventilation, extracorporeal membrane oxygenation, fiber optic bronchoscopy to clear sputum	E01, E02, E03, E04, E05, N01, D01
9, (53, male)	January 20	First admitted to The Third Affiliated Hospital of Sun Yat‐Sen University on January 20 and transferred to the ICU on January 23. Transferred to the ICU of The FAHGMU on February 3.	COVID‐19 (critical), acute respiratory distress syndrome (severe), respiratory failure, septic shock, myocardial injury, hypertension, sleep apnea syndrome, chronic hepatitis B	March 10‐11 (2 d)	57	Intubated ventilation, extracorporeal membrane oxygenation ventilation, fiber optic bronchoscopy to clear sputum	E01, E02, E03, E04, E05, N01, D01
10, (72, male)	January 25	First admitted to The Eighth People's Hospital of Guangzhou on January 25. Transferred to the ICU of The FAHGMU on February 3.	COVID‐19 (critical), acute respiratory distress syndrome (severe), acute kidney injury, myocardial injury, coagulation disorders, multiple organ dysfunction syndrome, diabetes, coronary atherosclerotic heart disease, hypertension, chronic obstructive pulmonary disease	March 12‐13 (2 d)	47	Non‐invasive ventilation, suction to clear sputum	E01, E02, E03, E04, E05, N01, D01

^a^ Frequencies and dates when these procedures were performed are described in Figure 2.

^b^ Refer to Table 1 for a description of the samples.

**Figure 3:**
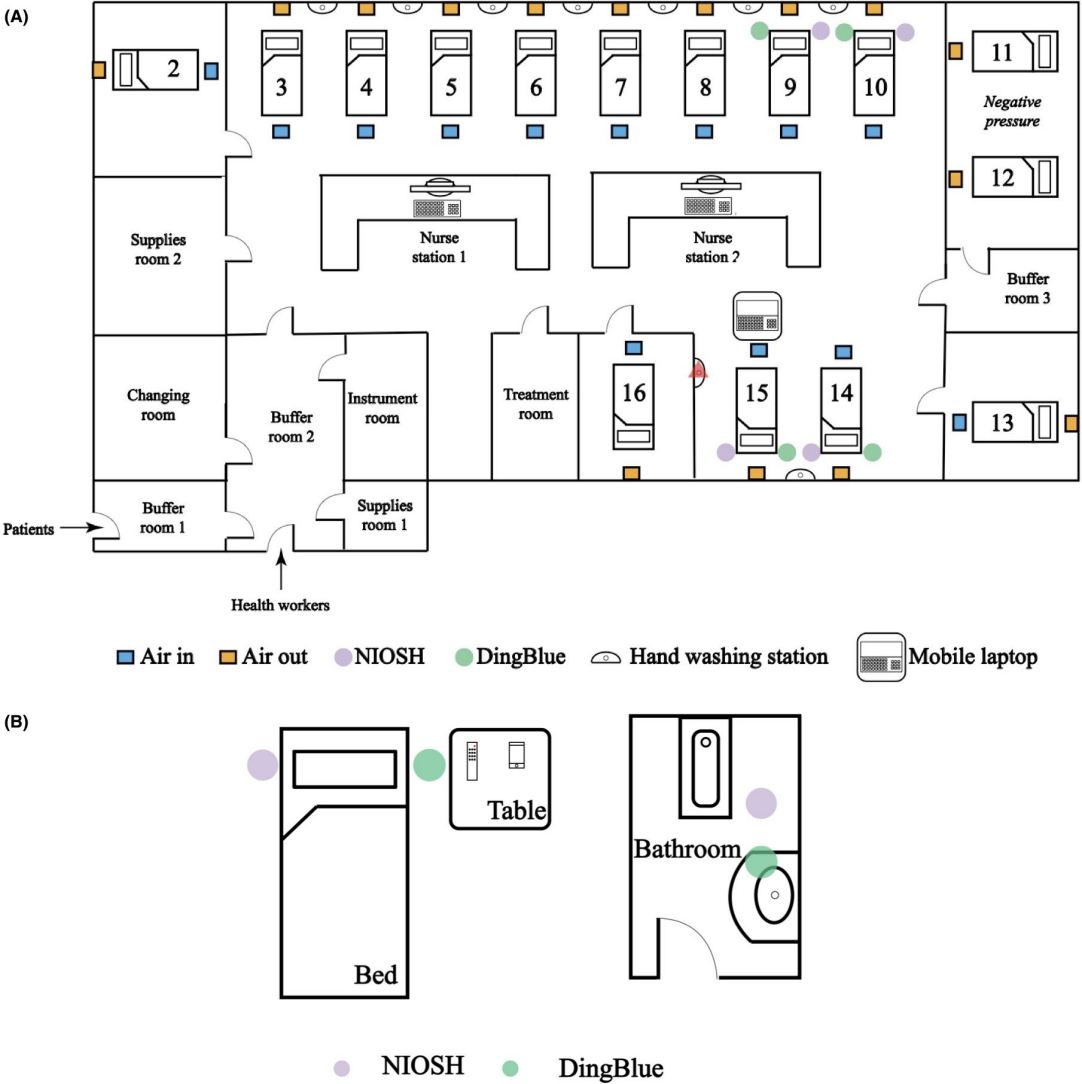
Layouts of hospital rooms. Samples were collected in the intensive care unit (ICU) (A) and in an isolation ward (B). In the ICU, airflow originated in the ceiling above the foot of each patient bed and was extracted through vents in the wall at bed height. Placement of the NIOSH and DingBlue air samplers and bed numbers are shown. The red triangle indicates the handwashing station that was sampled. In the isolation ward, each patient was isolated in different rooms with their own bathrooms. The locations of the NIOSH and DingBlue air samplers are shown in their positions relative to the patient's bed, toilet, and bathroom sink. Diagrams are not to scale. Refer to Table 1 for a list of air and environmental samples collected

No samples collected in the ICU were positive by qPCR based on the criteria set by the Sansure detection kit. However, one air sample collected using the DingBlue sampler placed near the head of the patient in bed 14 showed amplification at cycle threshold (Ct) 41.25. This patient already had serum antibody titers to the SARS‐CoV‐2 before this positive sample was collected and had subsequently developed virus neutralization antibody titers detected using a pseudoparticle assay after our study was completed (Table 5). Aside from the collection of respiratory samples and an anal swab, no other aerosol‐generating procedures were performed on the day that this sample was collected (Figure 3A). Overall, little environmental contamination with SARS‐CoV‐2 RNA was detected in the ICU.

### 3.2 Detection of environmental contamination with SARS‐CoV‐2 in the isolation ward

The population of the isolation ward consisted of five patients that had recovered from severe COVID‐19 but were still returning samples that tested positive for SARS‐CoV‐2 RNA. These patients had been hospitalized for 37, 39, 40, 45, and 47 days when we commenced sampling (Table 3). Unlike the ICU, patients in the isolation ward could move about and have access to their own bathroom. Therefore, we collected samples from patient rooms and bathrooms. The temperature, relative humidity, and concentration of CO_2_ in the ward over the sampling days were relatively constant, at 23.00 ± 0.32°C, 61.40 ± 13.29%, and 627.33 ± 83.35 ppm, respectively.

**Table 3:** Clinical, hospitalization history, and sampling details for patients in the isolation ward of The First Affiliated Hospital of Guangzhou Medical University

Patient Bed No, age, gender	Date of symptom onset	Hospitalization history	Admission diagnosis and symptoms exhibited during sampling period	Date of sampling (duration)	Duration of illness by time of sampling (days)	Samples collected
38, (58, male)	January 17	Admitted to The Eighth People's Hospital of Guangzhou on February 3, admitted to the intensive care unit (ICU) of The First Affiliated Hospital of Guangzhou Medical University (FAHGMU) on February 3, transferred to isolation ward on March 11	COVID‐19 (severe), occasional cough, a little phlegm, fatigue, applied non‐invasive ventilation between February 16 and 19	14‐15 (2 d), March 20 (1 d)	57	E01, E02, E04, E05, E06, E07, E08, E09, E10, E11, E12
18, (53, male)	January 24	Admitted to The Eighth People's Hospital of Guangzhou on January 30, admitted to the ICU of The FAHGMU on February 4, transferred to isolation ward on March 9	COVID‐19 (critical), cough, sputum	14‐15 (2 d), March 20‐23 (4 d)	50	E01, E02, E04, E05, E06, E07, E08, E09, E10, E11, E12, D01, D02, D03, N01
27, (82, female)	January 30	Admitted to, the isolation ward of The FAHGMU on February 6	COVID‐19 (severe), no symptoms noted	March 20‐23 (4 d)	54	E01, E02, E04, E06, E07, E08, E10, E11, E12, D02, D03
41, (50, male)	January 31	Admitted to The Second People's Hospital of Guangdong Province on February 6, transferred to the isolation ward of The FAHGMU on February 8	COVID‐19 (mild), no symptoms noted	14‐15 (2 d), March 20 (1 d)	43	E01, E02, E04, E05, E06, E07, E08, E09, E10, E11, E12
30, (26, male)	January 25	Admitted to the ICU of The FAHGMU on February 6 and transferred to the isolation ward on February 14.	COVID‐19 (severe), occasional cough, a little phlegm, pain in the right arm with bruising	March 21‐23 (3 d)	57	E01, E02, E04, E06, E07, E08, E10, E11, E12

Of the 182 samples collected over six days, three samples collected from the same patient (patient 18) were positive based on the standard diagnostic cutoff Ct of 40. The positive samples were the patient's facemask (Ct = 38.6) and two air samples collected in the bathroom on two different days (Ct = 35.6 and 35.5) (Table 4 and Figure S1). Using a less stringent cutoff (Ct < 45), two other samples collected from this patient; a facemask (Ct = 44.9) and an air sample collected in the patient's room (Ct = 44.7) were positive. Consistently, high viral loads were detected in lower respiratory tract (LRT) samples collected from this patient during the sampling period (9.8 × 10^4^, 7 × 10^7^, 2.4 × 10^6^, 6.3 × 10^8^, 1.7 × 10^7^, and 1.6 × 10^8^ copies/mL) but samples collected from the upper respiratory tract (URT) tested negative (Figure 3B). No stool samples were collected on the sampling days but samples taken two days prior to environmental sampling tested positive for SARS‐CoV‐2 RNA. An anal swab collected during the positive sampling day was negative (Table 4). This patient showed high serum IgG titers against SARS‐CoV‐2 S and N proteins and high virus neutralization antibody titers detected using a pseudoparticle assay before and after our sampling was conducted (Table 5).

**Table 4:** Details of air and surface samples with Ct value of <45 in the quantitative PCR assay

Ward	Sample ID	Date of sample	Patient	Sample information	Ct value	Viral load in patient samples (Ct value, RNA copy number/mL)^a^
Isolation	E07	March 14	18	Patient's facemask	38.6	LRT: 27.5, 9.8 × 10^4^; URT(P): negative
	D02	March 15	18	Bathroom air sample	35.6	LRT: 18.2, 7.0 × 10^7^
	D01	March 22	18	Bathroom air sample	35.5	LRT: 20.2, 1.7 × 10^7^
Samples with Ct value > 40						
ICU	D01	February 29	14	Air sample near bed	41.5	URT(P): 24.5, 8.3 × 10^5^; URT(N): 30.1,1.6 × 10^4^; LRT: 31.9, 4.5 × 10^3^, An: 39.2, 2.7 × 10
Isolation	E05	March 14	38	Patient's mobile phone	44.7	LRT: negative
	E01	March 14	41	Floor < 1 m from patient's head	42.4	URT(P): 32.9; 2.2 × 10^3^; LRT: weak; stool: negative
	E02	March 14	41	Floor > 1 m from patient's head	41.2	URT(P): 32.9; 2.2 × 10^3^; LRT: weak; stool: negative
	E07	March 15	18	Patient's facemask	44.8	LRT: 18.2, 7.0 × 10^7^
	D01	March 15	18	Air sample of the ward	44.6	LRT: 18.2, 7.0 × 10^7^
	E05	March 23	27	Patient's mobile phone	41.0	URT(N): negative; LRT: weak; plasma, stool, and urine: all negative

Note

Anal = anal swab; LRT = sputum/deep sputum; negative = Ct>40; suspicious = 38<Ct < 40; URT(N) = nasal swab; URT(P) = pharyngeal swab.

^a^ All samples that were collected for RT‐PCR testing on the day were listed.

**Table 5:** Serum IgG antibody titers and neutralizing antibody titers to SARS‐CoV‐2 pseudoparticles in patients in the intensive care unit and isolation ward of The First Affiliated Hospital of Guangzhou Medical University

Ward	Bed Number	Date	IgG antibody titer^a^		Neutralizing antibody titers^a^
			Spike protein (S)	Nucleocapsid protein (N)	
Intensive care unit	14	February 12	<100	3200	Not done^b^
		April 7	25 600	25 600	>1280
Isolation ward	18	February 12	102 400	102 400	>1280
		April 7	25 600	25 600	640
	27	February 12	<100	6400	Not done^b^
		April 7	6400	12 800	320
	38	February 12	25 600	102 400	40
		April 3	1600	102 400	40
	41	February 12	<100	<100	Not done^b^
		April 7	12 800	1600	40

^a^ Starting serum dilutions of 1:100 and 1:40 were used in ELISAs and microneutralization assays, respectively.

^b^ Samples testing negative for anti‐S IgG by ELISA were not tested by neutralization assay.

Four samples with Ct values >40 but <45 were collected from three other patients. Two samples were collected from the floor of a patient's room (Ct = 42.4 and 41.2). The viral load in the URT of this patient was 2.2 × 10^3^ viral copies/mL on this day. The remaining two samples were collected from the surface of the patient's mobile phones (Ct = 44.1 and 41.0, respectively). Further, URT and LRT samples taken from these patients tested negative for SARS‐CoV‐2 RNA. Two of the three patients had serum IgG titers to the SARS‐CoV‐2 S and/or N proteins before these samples were collected and one had neutralizing serum antibody titers detected using a pseudoparticle assay (Table 5). After sampling, all patients had serum IgG titers to the SARS‐CoV‐2 S and N proteins and neutralizing serum antibody titers detected using a pseudoparticle assay (Table 5). These findings suggest that persistently infected, clinically improved patients may still shed virus into the environment and URT samples may be poor indicators of shedding potential for these formerly severely ill patients.

## 4 DISCUSSION

The COVID‐19 pandemic has placed an extraordinary strain on health care worldwide. In addition to formidable workloads, medical personnel face significant psychological stress due to concerns of nosocomial exposure, particularly in light of worldwide shortages of personal protective equipment. The extended duration of detection of SARS‐CoV‐2 in patient swabs has meant that extended hospitalization and/or isolation are often required, sometimes for weeks, placing further strain on resources. While such patients test positive for SARS‐CoV‐2 RNA for extended periods, it is not known whether they could pose a risk of transmission. As such, we studied the environmental contamination associated with patients that had been hospitalized for weeks post‐COVID‐19 diagnoses. Due to the timeframe involved, these patients may also have some humoral immunity to SARS‐CoV‐2. Therefore, we also measured serum antibodies to determine whether their presence mitigated SARS‐CoV‐2 environmental contamination. This is particularly relevant as the presence of serum antibodies is being considered as a surrogate of viral clearance to allow people to return to work.

SARS‐CoV‐2 has been found in hospital settings, in patient wards and in the ICU.^1^, ^2^, ^3^ However, these studies were conducted in wards where newly diagnosed patients were being cared for. The focus of our study was to assess SARS‐CoV‐2 environmental contamination in hospital settings caring for patients with prolonged COVID‐19. Two broad types of patient were studied here: (a) Severely ill COVID‐19 patients in an ICU and (b) individuals whom have recovered from severe COVID‐19 have been discharged from the ICU, but must remain in the hospital under isolation as they are still testing positive for SARS‐CoV‐2 in swabs. The patients in this study had been hospitalized for as long as 57 days.

We did not find much evidence of SARS‐CoV‐2 contamination in the ICU, even though these patients, particularly patients 10 and 14, still had high viral loads and underwent procedures that are likely to generate aerosols. We did detect a high Ct value in one air sample, indicating that SARS‐CoV‐2 RNA may potentially have been present in the air. Taken together, this suggests that the infection control measures and practices of the ICU did not generate much environmental contamination, at least not within the limits of detection of our approach. This finding was in contrast to a report detecting greater contamination in the ICU compared to the general ward in the Huoshenshan Hospital in the outbreak epicenter in Wuhan.^1^ It is worth noting that the Huoshenshan Hospital was built in ten days specifically to treat COVID‐19 patients, while the FAHGMU is a well‐established reference hospital for respiratory diseases and has extensive experience in dealing with zoonotic viral pneumonias, including cases of avian influenza viruses,^12^, ^13^, ^14^, ^15^ MERS,^16^ and importantly, was a key hospital during the SARS outbreak in 2003.^17^ Therefore, the ICU and hospital infection control measures in FAHGMU are well established.

We detected more evidence of environmental contamination with SARS‐CoV‐2 RNA, including in the air, in the isolation ward. Positive samples were detected in the room and bathroom of one patient in particular. This patient, which had been severely ill since January 24, recovered and was transferred to the isolation ward on March 9. Samples taken from the LRT of this patient consistently tested positive for SARS‐CoV‐2 RNA (31/38 samples collected), as did stool samples (11/19 samples collected). The detection of viral RNA in the air samples in the bathroom suggests the virus‐laden aerosols are generated while using the bathroom. Notably, we also collected the bathroom air samples after an hour, and although there was some amplification signal, it was too weak to be included. The detection of viral RNA in air in the bathroom, along with evidence of frequent contamination of bathroom surfaces, indicates that infection could potentially occur even in the absence of direct contact with fecal matter.^2^ Therefore, sanitation of toilets should be emphasized, even in those who have recovered from severe illness.

Positive environmental samples were collected in the vicinity of patients that already had detectable serum antibody titers to the SARS‐CoV‐2 S and N proteins and neutralizing antibodies detected with a pseudoparticle assay. With COVID‐19 causing significant disruptions to economies worldwide, serum antibodies are being considered as a surrogate marker to determine the transmission risk. Here, we found that patients with serum antibodies against SARS‐CoV‐2 S and N proteins can still be associated with environmental contamination of SARS‐CoV‐2. As such, further studies may be required to further investigate the utility of serum antibodies as a surrogate marker of the ability of an individual to return to work safely.

Our study had several limitations. As we were unable to perform virus isolation, we cannot confirm the presence of viable viruses in our samples. With increasing Ct values, the likelihood of successful virus isolation and thus infectivity would likely decrease. Therefore, it is not known whether these samples could be capable of infecting a healthy individual. Due to the national biosafety regulation and limited resources during the outbreak, we were unable to sample more frequently or consistently. Lastly, the Ct values we detected in this study were in the lower limits of the detection threshold. This could be due to two potential factors; these patients had been infected for a long time and did not have high viral loads, particularly in the URT, or the heat inactivation step performed as part of the national biosafety requirement could have increased the Ct values of the samples.^18^ Therefore, we applied a less stringent Ct cutoff compared to the standard clinical diagnostic criteria to our qPCR results.

The discovery of SARS‐CoV‐2 RNA in air samples in the bathroom on multiple days suggests that virus‐laden particles are generated by toilet usage. Therefore, the risk of nosocomial infection with SARS‐CoV‐2 may be greater in bathrooms and in wards caring for mild patients compared to the ICUs, where we found minimal evidence of viral presence. Although the greatest transmission risks are still likely posed by patients early in infection when they have high viral loads, our study shows that infected patients may still pose a risk weeks after their initial diagnosis.^18^, ^19^ Further, as these patients had serum antibodies, further studies may be warranted to study the utility of serum antibodies as a surrogate of viral clearance. We recommend that healthcare workers continue to be vigilant even with patients who appear to have recovered.

## AUTHOR CONTRIBUTION

**Hui Lei:** Data curation (equal); Investigation (lead); Methodology (equal); Project administration (lead); Writing‐original draft (supporting); Writing‐review & editing (supporting). **Feng Ye:** Investigation (equal); Methodology (equal); Project administration (lead); Resources (lead); Writing‐original draft (supporting). **Xiaoqing Liu:** Investigation (equal); Methodology (equal); Project administration (lead); Resources (lead); Writing‐original draft (supporting). **Zhenting Huang:** Investigation (lead); Methodology (equal); Project administration (equal). **Shiman Ling:** Investigation (lead); Methodology (equal); Project administration (equal). **Zhanpeng Jiang:** Data curation (equal); Formal analysis (equal); Investigation (equal). **Jing Cheng:** Investigation (equal); Methodology (equal); Project administration (equal). **Xiaoqun Huang:** Investigation (equal); Methodology (equal); Project administration (equal). **Qiubao Wu:** Investigation (equal); Methodology (equal); Project administration (equal). **Shiguan Wu:** Investigation (equal); Methodology (equal). **Yanmin Xie:** Methodology (lead); Resources (lead); Supervision (supporting). **Cheng Xiao:** Investigation (equal); Writing‐original draft (equal). **Dan Ye:** Project administration (supporting); Resources (supporting). **Zifeng Yang:** Project administration (supporting); Resources (supporting); Supervision (equal). **Yimin Li:** Resources (supporting); Supervision (supporting). **Nancy Hiu Lan Leung:** Resources (lead); Supervision (supporting); Writing‐review & editing (equal). **Benjamin Cowling:** Methodology (equal); Resources (lead); Writing‐review & editing (equal). **Jianxing He:** Project administration (equal); Resources (equal); Supervision (equal); Writing‐review & editing (equal). **Sook‐San Wong:** Conceptualization (lead); Data curation (lead); Formal analysis (lead); Funding acquisition (supporting); Investigation (lead); Methodology (equal); Project administration (equal); Resources (lead); Supervision (lead); Writing‐original draft (lead); Writing‐review & editing (lead). **Mark Zanin:** Conceptualization (lead); Data curation (lead); Formal analysis (lead); Funding acquisition (lead); Investigation (lead); Methodology (lead); Project administration (lead); Resources (lead); Supervision (lead); Writing‐original draft (lead); Writing‐review & editing (lead).

## ETHICAL APPROVAL

The ethics committee of The FAHGMU has approved the use of patient's clinical data for this study (Ethics No. 2020‐65).

## Supporting information

Supplementary Material
