# Supplementary figures and images for: SARS‐CoV‐2 environmental contamination associated with persistently infected COVID‐19 patients
Source: Influenza Other Respir Viruses. 2020 Jul 12;14(6):688–99. doi: 10.1111/irv.12783 (PMC7361718; doi:10.1111/irv.12783)

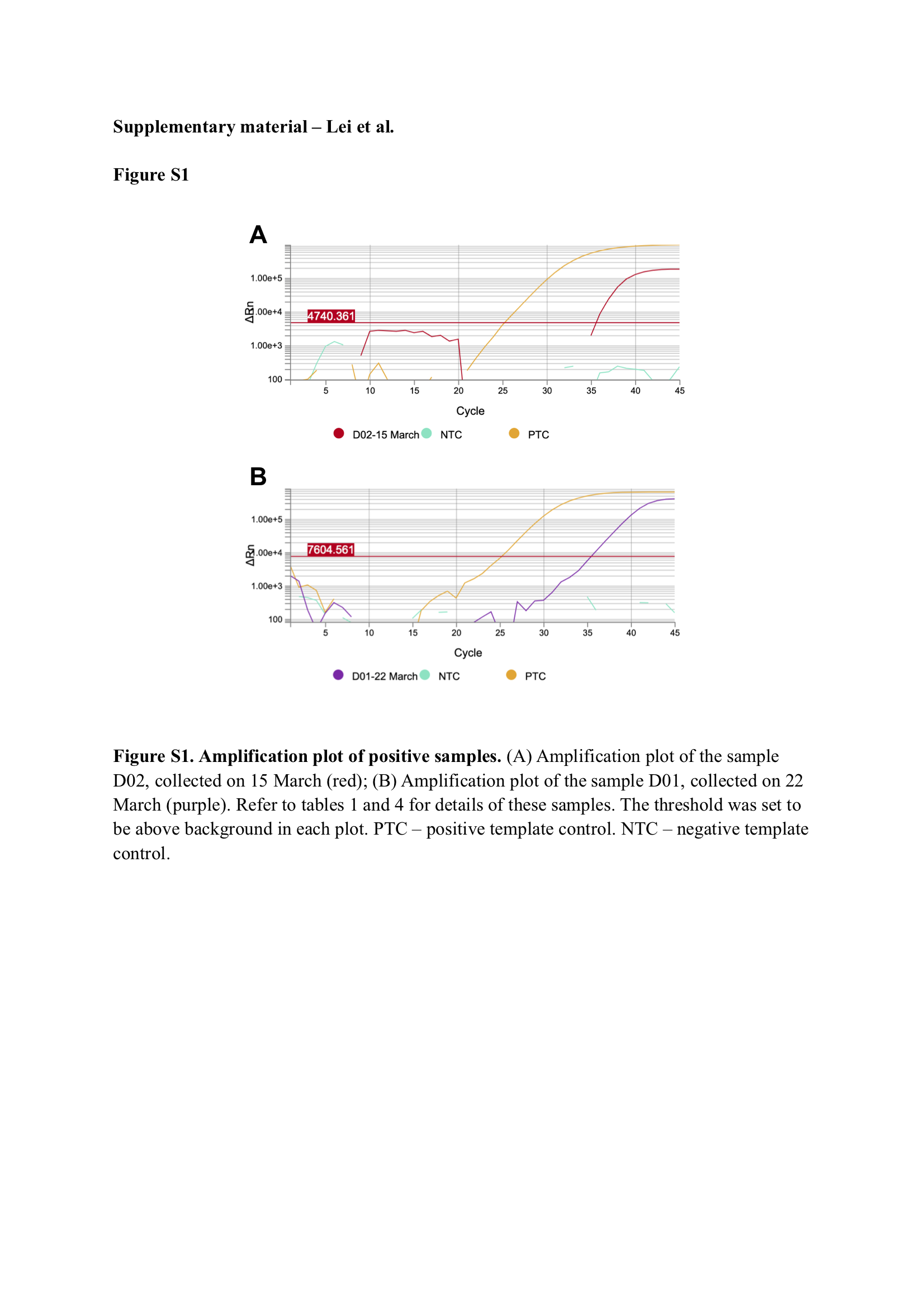

Supplement: Supplementary file 1 — Supplementary Material [file IRV-14-688-s001.tiff]
